# To Screen or Not to Screen Cutaneous Melanoma: A Case Report

**DOI:** 10.7759/cureus.92470

**Published:** 2025-09-16

**Authors:** Gloria S Kim, Lillian Chen, Jennifer Chew, Gurveen Sandhu, Amy S Wang

**Affiliations:** 1 Department of Medicine, University of California Los Angeles David Geffen School of Medicine, Los Angeles, USA

**Keywords:** cancer prevention, melanoma, screening guidelines, skin cancer, skin examination

## Abstract

As with all malignancies, the challenges of detecting and treating melanomas and other cutaneous malignancies at their earliest stages remain a priority. Routine skin examinations offer physicians an easy and cost-effective way to detect cutaneous melanomas and other skin cancers in high-risk individuals. However, there are no national consensus guidelines on skin cancer screening in the United States. Primary care physicians (PCPs) and dermatologists routinely face the challenge of when and how often to perform a total body skin examination (TBSE). Furthermore, PCPs are often hesitant to perform skin examinations due to limited training and a lack of confidence in proper diagnosis. This is a case report of a high-risk individual newly diagnosed with melanoma during an annual examination. This case will highlight the current issues and controversies surrounding screening guidelines for melanomas and non-melanoma skin cancers and the need to consider targeted screening in high-risk individuals.

## Introduction

Cutaneous skin cancers are the most common cancers diagnosed in the United States [1]. Of these, melanomas are associated with the worst survival outcomes. Current five-year survival rates for melanoma are 98.4% for localized disease, 63.6% for regional disease, and 22.5% for metastatic disease [2]. It is estimated that the number of new cases of melanoma diagnosed in the United States in 2025 will increase by 5.9% with the most common types being superficial spreading melanoma (70%), followed by nodular melanoma (20%) and acral lentigo melanoma [3]. Superficial spreading tends to grow more slowly and horizontally, while nodular can have a quicker, more deeply invasive pattern, thereby being associated with a worse prognosis [4].

Currently, the lifetime risk of getting melanoma is about 3% (one in 33) for Caucasians, 0.1% (one in 1,000) for African-Americans, and 0.5% (one in 200) for Hispanic people. Risk factors include obvious issues such as lighter skin, UV exposure, and dysplastic nevi, but can also include male sex, immunodeficiency, age, family history, and number of moles (>50 nevi) [5]. Due to the rising incidence of skin cancers, especially in high-prevalence areas such as Southern California, PCPs often serve as the first point of contact for screening and diagnosis. As with all malignancies, early detection may improve morbidity and mortality, especially in those with a more aggressive subtype, such as melanoma, especially in high-risk individuals described above. 

In the United States, it is estimated that treatment of skin cancers comes at an annual cost of approximately $8.1 billion, $3.3 billion of which is related to melanoma specifically [6], with many of these costs likely to increase with the usage of expensive drug therapies like immunotherapy implemented for more advanced stage disease. Moreover, because of the morbidity and mortality complications associated with skin cancers, specifically with melanoma, the idea of surveillance for early detection has been an ongoing debate.

TBSE has the potential to identify early-stage melanomas and other non-melanoma skin cancers before they progress to higher-stage disease with increasing morbidity and mortality. There are already nationwide public health screening initiatives like the SPOTMe® program advocated by the American Academy of Dermatology (AAD). Studies have demonstrated that implementation of a structured surveillance program can be performed with high efficiency in high-risk patients [7]. Moreover, some studies have correlated earlier detection of melanoma with improving long-term absolute survival by almost 8%, especially with formal dermatologic evaluation [8].

However, accepting the efficacy of screening has been challenging due to the lack of large prospective randomized controlled trials to study the diagnostic impact of screening and its impact on mortality. Due to the lack of national guidelines, it is not surprising that routine screening practices remain inconsistently practiced amongst frontline physicians.

## Case presentation

**Figure 1:**
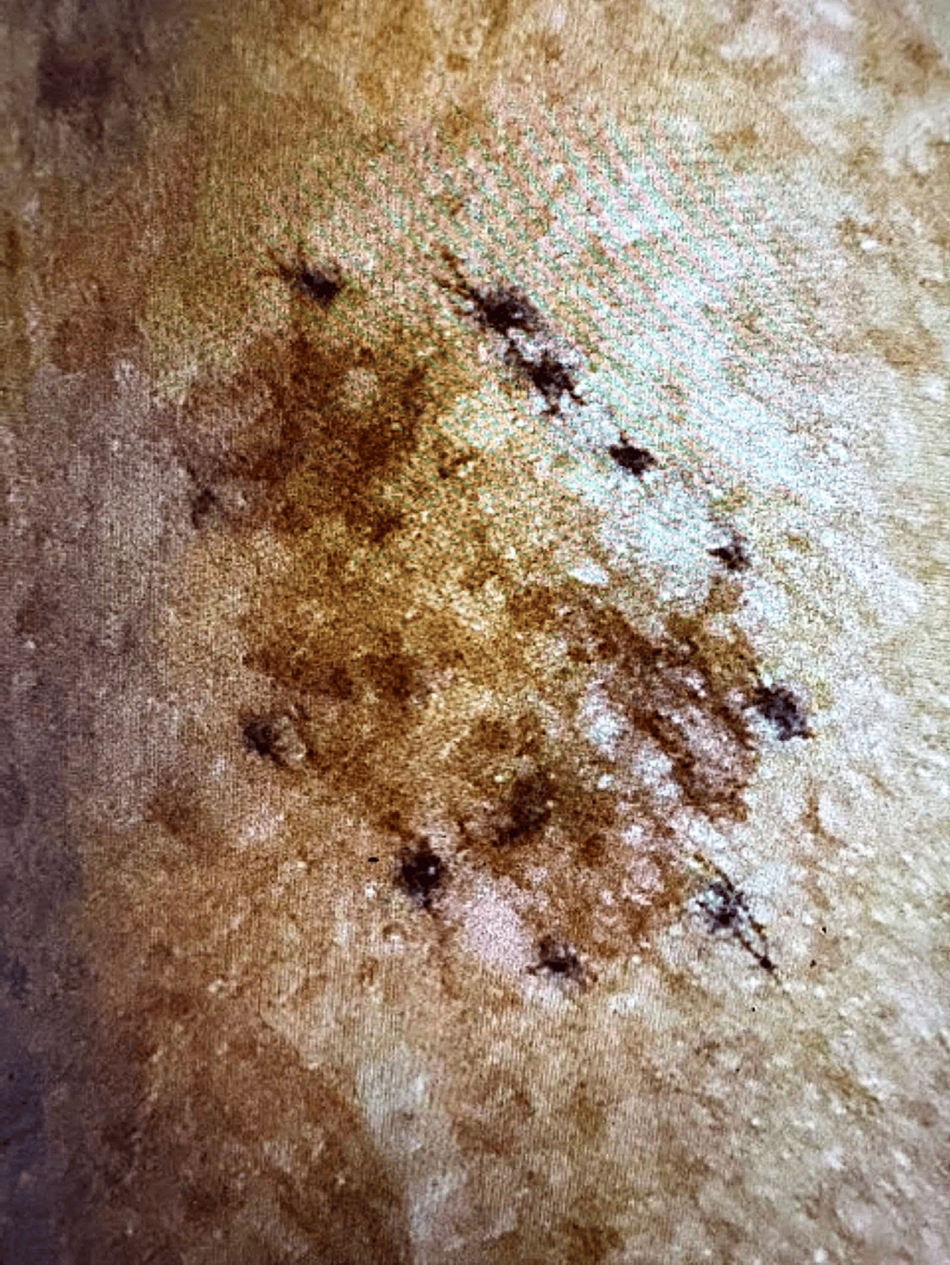
Suspicious skin lesion on the left posterolateral upper arm with irregular borders and pigmented variation biopsied to be melanoma, superficial spreading subtype.

A 71-year-old male with a past medical history of hypertension and hyperlipidemia presented for a routine annual physical examination. On examination, a pigmented lesion with irregular borders and asymmetry was noted near his left posterolateral upper arm (Figure 1).

He reported no symptoms of this lesion and had not noticed it previously. He endorsed extensive lifetime sun exposure while working as a lifeguard for over 20 years. His dermatologic history included actinic keratosis treated with cryotherapy and basal cell skin cancer of the upper back treated with Mohs procedure eight years prior. His estimated five-year risk of developing melanoma was approximately 9% by the National Cancer Institute Melanoma Risks Assessment Tool [9]. His family history was nonspecific. Physical examination revealed numerous clinically appearing solar lentigines and small actinic keratoses in sun-exposed areas of his body. The lesion in question measured approximately 7-8 mm with some irregularity of the borders and color variation. No lymph nodes were palpable in the left axilla. Laboratory studies, including a complete blood count and comprehensive metabolic panel, were normal except for a mildly elevated total bilirubin level at 1.5 mg/dL (Table 1).

**Table 1:** Laboratory values

		Reference range
Sodium	141	135-146 mmol/L
Potassium	4.7	3.6-5.6 mmol/L
Chloride	106	96-106 mmol/L
Total CO_2_	26	20-30 mmol/L
Anion gap	9	8-19 mmol/L
Glucose	87	65-99 mg/dL
Creatinine	0.73	0.60-1.30 mg/dL
Urea nitrogen	13	7-22 mg/dL
Calcium	9.3	8.6-10.4 mg/dL
Total protein	6.7	6.1-8.2 g/dL
Albumin	4.4	3.9-5.0 g/dL
Total bilirubin	1.5	0.1-1.2 mg/dL
Alkaline phosphatase	73	37-133 U/L
AST (SGOT)	32	13-62 U/L
ALT (SGPT)	19	8-70 U/L
White blood cell count	7.53	4.16-9.95 x 10E3/uL
Red blood cell count	4.56	4.41-5.95 x 10E6/uL
Hemoglobin	13.9	13.5-17.1 g/dL
Hematocrit	42.9	38.5-52%
Platelet count	230	143-398 x 10E3/uL

The patient was sent for dermatologic evaluation, where a punch biopsy of the lesion revealed a melanoma, superficial spreading subtype. Of note, dermoscopy (a noninvasive method that allows the in vivo evaluation of colors and microstructures of the epidermis, the dermoepidermal junction, and the papillary dermis not visible to the naked eye) was not performed. The tumor measured 0.9 mm in Breslow thickness and demonstrated ulceration. Mitotic index was 2/mm^2^. No lymphovascular invasion or microsatellites were noted. He subsequently underwent a wide excision and sentinel lymph node biopsy showing microscopic involvement of one lymph node without in-transit, satellite, or microsatellite metastases. The primary tumor measured 0.9 mm in thickness with ulceration. One centimeter margins were achieved.

FDG-PET/CT scan showed no evidence of distant metastasis, and he was staged as IIIA (T1b N1a M0). The patient has since been referred for oncologic evaluation for recommendations on the need for adjuvant treatment.

## Discussion

Understanding the prevalence of skin cancers and the aggressiveness of melanomas, one would logically conclude that from a public health standpoint, implementation of skin cancer screening guidelines, especially in high-risk individuals, either from physician examination or education for patient self-examination, would garner benefits in terms of prevention of cancer-associated morbidity and mortality effects. Such low-cost practices are already used for other common malignancies, for example, digital rectal examinations in men for prostate cancer and self-breast examinations in women for breast cancer. Visual skin cancer screening in high-risk patients has been shown to be possibly associated with an increase in discounted life expectancy while reasonably cost-effective [10].

However, many barriers currently exist to implementing an effective screening program. Currently, it is estimated that only approximately 8% of patients who have seen a PCP have received a skin examination [11]. A lack of adequate training in skin cancer detection, competing priorities, and time constraints make TSBEs challenging in the primary care setting. In addition, access to proper care remains an issue with underinsured and uninsured patients. Studies have shown a correlation between various social determinants of health and the presentation of melanoma at more advanced stages, which is associated with higher mortality rates [12]. Furthermore, the United States Preventive Services Task Force currently reports insufficient evidence to recommend routine skin cancer screening (Table 2) [13].

**Table 2:** United Stated Preventative Services Task Force (USPSTF) recommendations for skin cancer screening. Citation: [9]

Rationale	Assessment
Detection	The USPSTF found adequate foundational evidence that visual skin examination by a clinician has modest sensitivity and specificity for detecting melanoma. However, skin cancer has primarily been studied in persons with fair skin, so the evidence may not be applicable to all skin colors. Evidence is limited regarding the accuracy of the clinical visual skin examination for detecting keratinocyte carcinoma.
Benefits of early detection and intervention and treatment	The USPSTF found inadequate evidence that screening for skin cancer through skin examination by a clinician reduces morbidity or mortality.
Harms of early detection and intervention and treaent	The USPSTF found inadequate evidence of the harms of skin cancer screening and diagnostic follow-up.
USPSTF assessment	Due to a lack of available data applicable to a US population, the USPSTF found that the evidence is insufficient to determine the balance of benefits and harms for visual skin examination by a clinician to screen for skin cancer in asymptomatic adolescents and adults.

Other professional organizations like the American College of Physicians, the National Cancer Institute, and the American Society of Clinical Oncology also do not offer specific guidance regarding skin cancer screening. Some of the concerns cited include the increase in detection of basal cell carcinomas in adults, which would have a limited impact on life expectancy [14]. Screening guidelines are often based on a demonstration of a reduction in mortality. However, designing studies to detect such outcomes would be extremely difficult and costly, as evidenced by the lack of such studies that have been undertaken.

By contrast, some other countries with similarly high incidences of skin cancers have adopted more general screening guidelines. Germany offers routine TSBE in all adults aged 35 or older every two years [15]. However, results from such a program are unclear, as one study shows no measurable decline in mortality due to melanoma since the introduction of skin cancer screening in Germany in 2008 [15]. The National Health and Medical Research Council of Australia recommends targeted screening every 6 months for high-risk patients assessed by skin/hair color, sun sensitivity, number of common and atypical nevi, chronic actinic skin damage, and family history of melanoma.

In this patient, a routine annual physical exam led to what was ultimately diagnosed as stage IIIA melanoma. Even though it is unknown whether this improved the patient’s overall prognosis, this detection allowed for early intervention, which is especially important given the decrease in survival rate even from IIIA to IIIC (93% vs. 69%) [16]. This case is an example of how targeted screening in high-risk individuals by PCPs, as well as the individual, can be a quick, cost-effective method of early detection, yielding potentially life-saving benefits.

## Conclusions

The question of the possible mortality reduction associated with melanoma screening outweighing the potential costs and harms of overdiagnosis remains unanswered. Due to the lack of skin cancer screening guidelines, PCPs are left to tailor their approach to the individual patient. However, as the number of new cases continues to rise, physicians may find that moving forward, the balancing act may need to tip in favor of some form of screening mechanism, especially in the high-risk population. In the end, we recommend that PCPs consider TBSEs in high-risk individuals despite the lack of consensus guidelines.
